# Orphan Applicants in Orthopedic Surgery: Where Do Allopathic Applicants Without an Affiliated Residency Program Match?

**DOI:** 10.7759/cureus.64343

**Published:** 2024-07-11

**Authors:** Daniel I Razick, David Chen, Akash Pathak, Jimmy Wen, Mouhamad Shehabat, Austin Lee, Carter Bernal, Muzammil Akhtar, Amir A Jamali

**Affiliations:** 1 Surgery, California Northstate University College of Medicine, Elk Grove, USA; 2 Physical Medicine and Rehabilitation, California Northstate University College of Medicine, Elk Grove, USA; 3 Orthopedic Surgery, Joint Preservation Institute, Sacramento, USA

**Keywords:** freida, nrmp match, home program, orthopedic surgery, residency

## Abstract

Background

Orthopedic surgery is one of the most competitive specialties to match into a residency. With a plethora of qualified applicants and the subjective nature of matching into any residency program, it can be difficult to accurately assess the chances of successfully matching into orthopedic surgery and the types of programs an applicant will match into. The purpose of this study is to compare the types of programs that students from medical schools with and without home programs match.

Methods

This was a five-year retrospective study (2019 to 2023) analyzing 155 United States Doctor of Medicine (M.D.) programs and their orthopedic residency-matched students. Of the 155 programs, 40 were excluded from the study due to the lack of obtainable data. For each medical school, we analyzed several variables: the presence of a home program, the total number of orthopedic residency matches, residency program matches, and residency program affiliation (academic, community, university-affiliated community-based, military).

Results

Of the 2066 total matched applicants from institutions with home programs, 1508 (73%) matched into academic centers, 315 (15.3%) into university-affiliated community programs, 172 (8.3%) into community programs, and 71 (3.4%) into military programs. In contrast, of the 219 total matched applicants from institutions without home programs (orphan applicants), 144 (67.8%) matched into academic programs, 36 (16.4%) into university-affiliated community programs, 28 (12.8%) into community programs, and 11 (5%) into military programs.

Conclusion

A greater proportion of students from institutions with home programs matched into academic centers compared to orphan applicants (73% vs. 65.8%). A greater proportion of orphan applicants matched into community programs (12.8% vs. 8.3%).

## Introduction

Orthopedic surgery is one of the most competitive specialties to match into a residency [1]. According to the 2023 National Resident Matching Program data, 1,425 medical students applied for 899 available orthopedic surgery residency positions [2]. With the known competitiveness of applying to orthopedic surgery residencies, the number of applicants does not reflect applicants who self-screened themselves from applying to this specialty in the first place.

In a survey of orthopedic surgery program directors polled about the change of the United States Medical Licensing Examination (USMLE) Step 1 to pass/fail, most agreed that Step 2 will have greater importance. In their estimation, factors such as personal knowledge of the applicant and letters of recommendation from recognizable orthopedic surgeons would likely be important in granting interviews [3,4,5,6]. Around 58.7% of respondents additionally believed that allopathic students from highly regarded schools would have an advantage in their applications [3]. With a plethora of qualified applicants and the subjective nature of matching into any residency program, it can be difficult to accurately assess the chances of successfully matching into orthopedic surgery.

In this type of environment, students who do not have an affiliated home residency program might anticipate difficulties in establishing academic mentorship, developing professional relationships, accessing clinical and research opportunities, and receiving letters of recommendation. Shahriari et al. examined applications to programs in integrated plastic surgery, another competitive specialty, specifically in the context of “orphan applicants,” who are applicants with no associated home residency program [7]. They found that graduates from schools without home programs and international medical graduates represent a small minority of successfully matched students [7]. The purpose of the present study is to examine the demographic characteristics of so-called "orphan program applicants" in orthopedic surgery, to evaluate the program types to which these applicants match, and to compare these applicants to those allopathic students from medical schools with home programs.

## Materials and methods

The study was conducted at California Northstate University College of Medicine in Elk Grove, California. A five-year retrospective study (2019 to 2023) was conducted, analyzing 155 United States Doctor of Medicine (M.D.) programs and their orthopedic residency-matched students. Data was obtained through the Offices of Student Affairs (OSA) and publicly available residency match lists published on official medical school websites.

We examined 155 US M.D. programs for characteristics including the presence of a home orthopedic residency program and the number of orthopedic physician faculty (M.D. or D.O.) in the specialty. For each year (2019 to 2023) we obtained, if available, the total number of students that matched into an orthopedic residency program (#), the percentage of orthopedic surgery matches (%), the residency program the student matched into, and whether the residency program they matched into was academic, community, both (university-affiliated community programs), or military-based. Communications were sent to each M.D. program’s OSA requesting the aforementioned data through e-mail. No response or data were received from any of the 155 programs. Of the 155 programs, 40 were excluded from the study due to having no data from the program’s official website or response from the M.D. program’s OSA.

Home program and number of orthopedic physician faculty

Information on whether an M.D. program had a home orthopedic residency program was obtained through the medical school's official website. If at the time of data collection (July 2023) the program had an orthopedic residency program, then the program was recorded as having a residency program. At the time of data collection, the number of orthopedic physician faculty, both M.D. and D.O. physicians, was obtained using the same method and recorded.

Total number of orthopedic residency matches, residency program matched into

The total number of students that matched into orthopedic surgery was counted from each available match list. Match lists were found on the official medical school websites. If a medical school not only listed the number of students that matched into orthopedic surgery but also the precise names of the programs, this information would also be collected.

Residency program affiliation

Information on the type of residency program was collected from the Fellowship and Residency Electronic Interactive Database (FREIDA) tool provided by the American Medical Association [8]. For each matched student’s residency program, it was recorded as either academic, community, both (university-affiliated community programs), or military-based.

## Results

**Figure 1:**
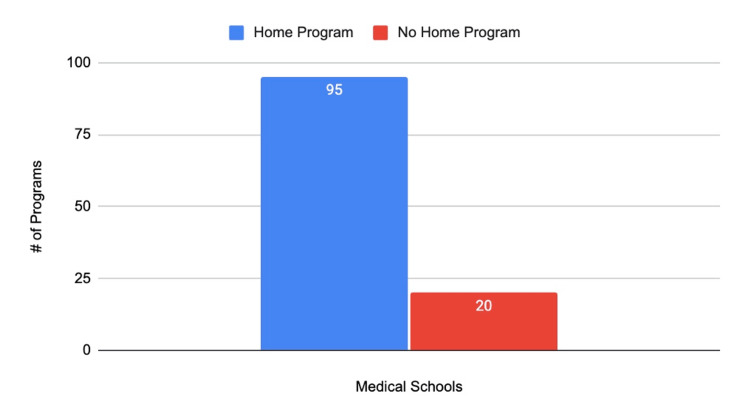
Number of U.S. MD schools with and without home programs

Of the US M.D. programs with match data available between 2019 and 2023 [8], 95 institutions had an affiliated home orthopedic surgery program, while 20 did not. The remaining statistics presented in subsequent figures should be viewed with the understanding that there are nearly five times as many institutions with home programs as there are programs without (Figure 1).

**Figure 2:**
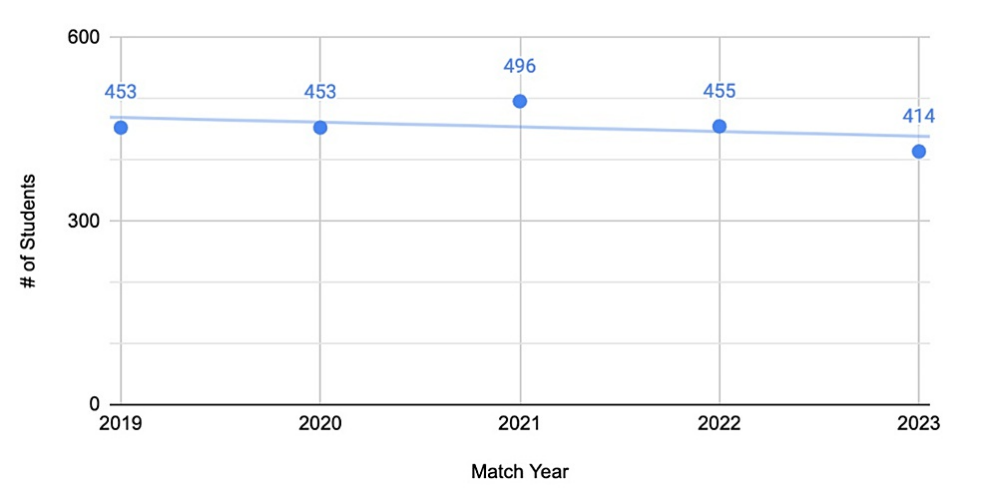
Total number of students who matched from medical schools with home programs (2019 to 2023)

**Figure 3:**
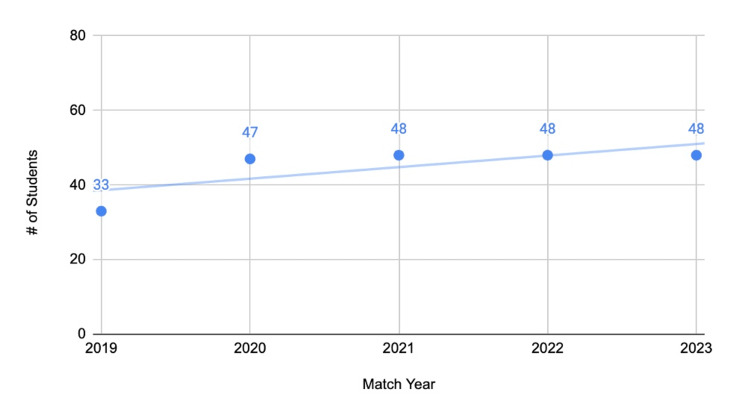
Total number of students who matched from medical schools without home programs (2019 to 2023)

From 2019 to 2023, an average of 454 students who attended medical schools with home programs matched into orthopedic surgery. Taking the average of students who matched compared to the number of schools with home programs (454/95), an average of 4.8 students matched per school (Figure 2). This number is highly variable from school to school and is only presented to compare to the statistics presented in Figure 3.

From 2019 to 2023, an average of 45 students who attended medical schools without home programs matched into orthopedic surgery. Taking the average of students who matched compared to the number of schools with home programs (45/20), an average of 2.25 students matched per school (Figure 3). This number is highly variable from school to school and is only presented to compare to the statistics presented in Figure 2.

**Figure 4:**
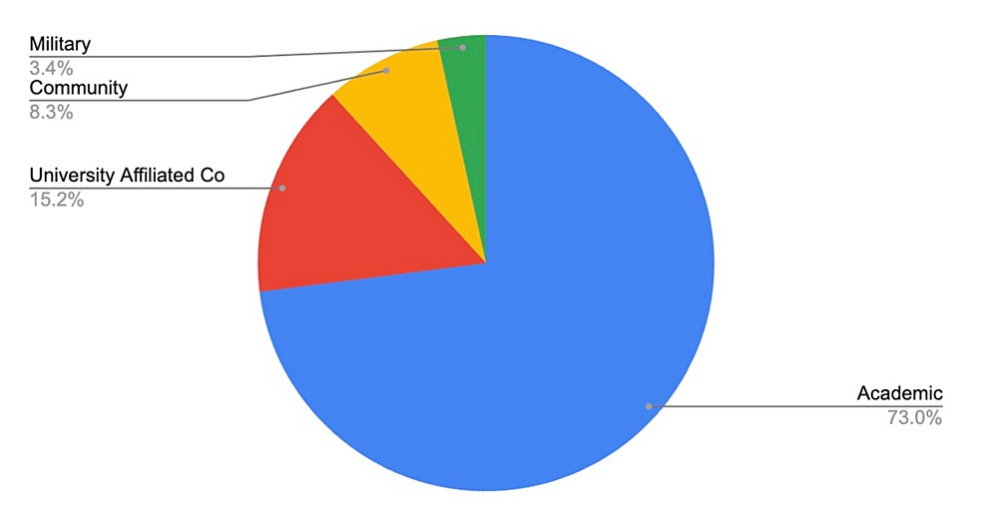
FREIDA program type match rates for applicants with home programs FREIDA: Fellowship and Residency Electronic Interactive Database

An overwhelming majority of applicants from medical schools with home programs matched at academic centers, while 15.2% matched at university-affiliated community centers, 8.3% at community programs, and 3.4% at military programs (Figure 4).

**Figure 5:**
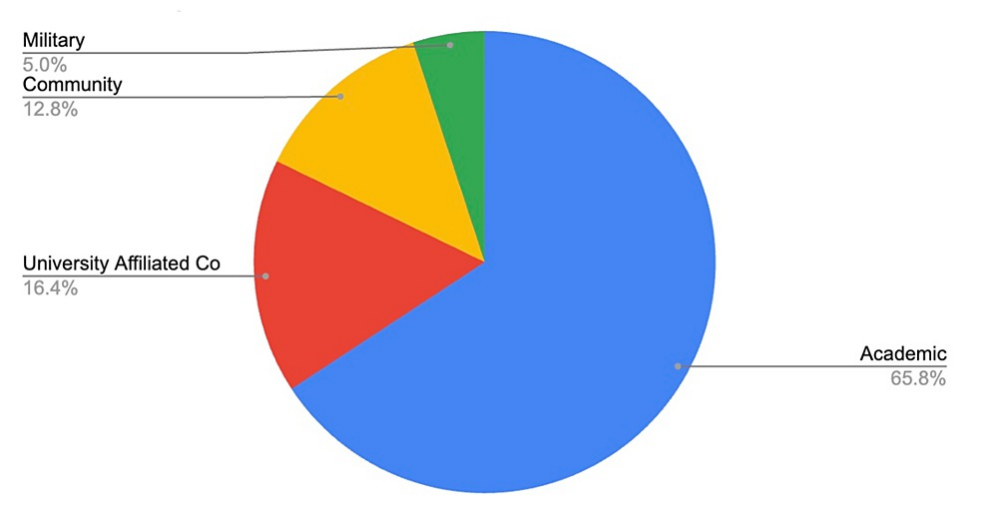
FREIDA program type match rates for applicants without home programs FREIDA: Fellowship and Residency Electronic Interactive Database

Compared to applicants from medical schools with home programs, applicants without home programs had similar match rates with regard to FREIDA program type. Around 65.8% of applicants matched at academic programs and 16.2% at university-affiliated community centers. Notably, there was a 4% increase in the number of community program matches and a 1.6% increase in military program matches, compared to applicants with home programs (Figure 5).

## Discussion

The primary objective of this study was to compare the match data for orthopedic surgery applicants from allopathic medical schools with and without home programs. The most important finding of the study is that of the 115 medical schools included in the study, nearly five times as many schools had affiliated home programs as those that did not. The breakdown of programs where students matched based on the FREIDA tool was similar for applicants with and without home programs, with over 65% matching into academic centers. An average of 4.8 students match into orthopedic surgery when applying from institutions with home programs, compared to an average of 2.25 from institutions without home programs. It should be noted that this figure is highly dependent on class sizes, and it is possible that institutions without home programs have smaller class sizes.

The United States currently has 155 Liaison Committee on Medical Education (LCME)-accredited allopathic medical schools, many of which have been in existence for over three decades, the oldest being the Perelman School of Medicine at the University of Pennsylvania, dating back to 1765 [9]. Many institutions have had sufficient time to construct medical centers or become affiliated with local hospitals to create residency programs, which could explain why there are nearly five times more schools with affiliated orthopedic surgery residencies than schools without. While not a primary finding of this study, the data indicated that between 2019 and 2023, an average of 17.5% of applicants matched at their home programs, with 2021 having the highest amount of internal matches at nearly 25%. These findings are consistent with trends seen in other competitive specialties such as plastic surgery (18.2%) and otolaryngology (20%) [7,10]. Due to the coronavirus disease (COVID-19) pandemic, 2021 was the first year in which virtual interviews were conducted, and it is plausible that programs selected students from their medical schools due to the familiarity of students before the application season. Given the limitations regarding sub-internships and away rotations, external candidates had fewer opportunities to showcase their abilities and impress attendings and program directors at other institutions. Nevertheless, just over 75% of students matched externally, indicating applicants managed to leave an impression on program directors despite the challenging and unprecedented circumstances.

The FREIDA tool provided by the American Medical Association (AMA) allows candidates to view comprehensive information regarding a residency program at the click of a button [8]. Our study utilized this tool to distinguish residency programs by type: academic, university-affiliated community, community, or military. Interestingly, the trends were similar for both students with and without home programs, with the overwhelming majority of students matching at academic centers. However, students without home programs matched at academic centers at an 8% lower rate than their home program counterparts and matched at community centers at a 4% higher rate. On average, over twice as many students are matched into orthopedic surgery per school with a home program compared to those without one. There are several potential explanations for this finding. Studying medicine at an institution with a home orthopedic surgery residency offers many advantages not available to students at institutions without home programs. Access to orthopedic surgeons can open the door to meaningful mentorship experiences, such as shadowing surgeons in the clinic or operating room and getting a better feel for the day-to-day life of an orthopedic surgeon [11]. For programs with faculty inclined toward basic science or clinical research, there is often a pipeline for medical students to pick up research projects that may eventually lead to a summer internship or research gap year, culminating in national presentations or peer-reviewed publications. Finally, getting to know the members of the home orthopedic faculty can lend itself to stronger and more intimate letters from these surgeons, which can lead to a substantial boost in the strength of the application for those students.

Academic hospitals have an expectation to not only provide excellent medical care but to continue innovating and moving the field of medicine forward [12]. Academic centers may favor applicants with greater research experiences, and these students may have attended schools where research opportunities were plentiful as a result of being affiliated with a home program. Community centers, where research may not be the primary focus, may place less emphasis on research and hence accept students with less research experience. Given the currently available data, it is unclear exactly how much research experience applicants from medical schools, with and without home programs, had when applying. The field of medicine has always been reserved for many of the most accomplished candidates. The current data indicate that applicants for competitive residency programs such as orthopedic surgery are at an advantage if they come from programs with an affiliated home orthopedic residency. This would suggest that matching in orthopedics may be dependent not only on academic achievements but also on relationships with strategically placed mentors in the field. Having a luminary surgeon from a well-known residency program send an email or make a phone call on an applicant’s behalf may have a substantial effect on their ultimately matching into residency [13].

Given the current data, it is difficult to say how many applicants had a personal connection to the institution where they matched, but future research should be geared toward understanding the intangible aspects of a competitive application. With USMLE Step 1 being pass/fail, program directors have lost a significant metric that could be used to distinguish among the top medical students in the application pool [6]. As a result, other factors, such as those mentioned above, may continue to hold more weight. Future research should be directed toward determining which variables are held in the highest regard and discovering the percentage of students who are taking research years prior to applying to orthopedic surgery.

Limitations

The findings of this study should be interpreted through the lens of its limitations. The primary limitations of this study are mostly due to the method of data collection and data availability. Data were compiled using program websites, and attempts were made at contacting medical school Offices of Student Affairs (OSA). Only data that was publicly available from official medical school sources was included. Some institutions published match data in some years and did not in others, while the programs that were excluded from the study did not have any match data published over the five-year period. Furthermore, several schools published the number of students who matched in a specific specialty but did not provide the exact programs in which their students matched. Therefore, we were unable to determine the type of program (academic, university-affiliated, community, or military) for those students and include them in the figure regarding program type. Due to the aforementioned limitations, the overall match information for orthopedic surgery is not entirely reflected in the present study. Variables such as graduate degrees, the presence of research, research years, volunteering, leadership, and intangibles such as personal connections are not taken into consideration in this study. However, certain trends remained consistent from year to year and have been presented.

## Conclusions

Applicants from institutions with and without home programs match into university-affiliated community centers at similar proportions, while a greater proportion of applicants from institutions with home programs match into academic centers. However, there are significantly more medical schools in the US with home programs than without, so the data in the present study should be interpreted with this fact in mind. This study highlights the advantages held by students from medical schools with home orthopedic programs in achieving their dream of matching into orthopedic surgery. A future investigation is warranted regarding tangible and intangible variables that play a role in the preparation of students applying to orthopedic surgery with and without home programs. Factors such as mentorship, research opportunities, and clinical exposure may play a significant role in aiding students match into orthopedic surgery. In spite of this non-level playing field, it is encouraging that, with the right planning and strategies, a substantial number of applicants from schools without a home program are still able to match into orthopedic surgery. Students should remain encouraged, regardless of the presence of a home program, that matching into a competitive field like orthopedic surgery is possible. 
